# Statistical Study of the Process Parameters for Achieving Continuous Consolidation of a Thermoplastic Composite

**DOI:** 10.3390/ma16206723

**Published:** 2023-10-17

**Authors:** Daniel Campos, Pere Maimí, Alberto Martín

**Affiliations:** 1 AMADE-UdG Research Group, University of Girona, 17003 Girona, Spain; pere.maimi@udg.edu; 2 Applus+ Laboratories, 08193 Bellaterra, Spain; alberto.martin@applus.com

**Keywords:** thermoplastic materials, LM-PAEK, PEEK, consolidation, manufacturing process

## Abstract

Manufacturing components using thermoplastic composite materials necessitates a judicious balance among fabrication parameters, cost considerations and the ultimate quality of the elements produced. Continuous manufacturing technologies, exemplified by methods such as continuous compressing molding and glide forming, seek to revolutionize production through their continuous processing approach. This study aimed to investigate the effects different process parameters have on the final quality of the manufactured parts when a continuous manufacturing technology, such as glide forming, is applied to thermoplastic composite materials. An experimental rig was designed, and 19 samples were prepared using a unidirectional-carbon-fiber-reinforced LM-PAEK (low-melting polyaryletherketone) composite. The process parameters studied were temperature, pressure and forming speed. The quality of the final parts was evaluated based on their thickness and consolidation levels. The findings underscore the feasibility of leveraging continuous manufacturing technologies for producing components using thermoplastic composite materials, but the process parameters must be carefully controlled to ensure the quality of the final part. The models obtained could be used as a post-processing tool to predict thickness and consolidation levels when simulating the manufacture of a component on macroscale levels. Further research is needed to optimize the process parameters and study their effects on other thermoplastic composite materials.

## 1. Introduction

Thermoplastic composites are the combination of fibers (such as glass, carbon or aramid) and a thermoplastic polymer matrix. The resultant is a lightweight material that combines the strength and resistance of the fibers with the malleability and chemical resistance of the polymer. Unlike thermoset polymers, thermoplastics consist of long chains of molecules that can be repeatedly softened and hardened through heating and cooling cycles without undergoing any chemical changes [1]. Thus, thermoplastics composites can be reshaped multiple times when heated making them more versatile than thermoset composites, enabling easier manufacturing and repair processes, as well as the possibility of recycling [2]. Thermoplastic composite materials have gained significant attention in the field of advanced materials due to their unique combination of high strength, low weight and the ability to be molded and reshaped under heat [3]. One important quality asset in the manufacturing of thermoplastic composite components is consolidation, which refers to the removal of excess resin and the creation of a uniform, dense structure within the composite layout. This phenomenon has a significant impact on the microstructure and porosity of the composite material, which in turn plays a crucial role in determining the final mechanical and physical properties of the composite material [4].

In recent years, there has been a significant amount of research into the optimization of thermoplastic composite consolidation processes. For example, Aquier et al. [5] reviewed the consolidation of unidirectional-carbon-fiber-reinforced LM-PAEK (low-melting polyaryletherketone) composites. They studied different fabrication techniques and the effects their parameters had on consolidation and mechanical properties. Colton et al. [6] investigated the processing parameters for consolidating PEEK (polyetheretherketone)/carbon fiber (APC-2) composites on resin flow measured by the Kozeny constant and the bulk modulus. They found that these variables depend on the consolidation pressure, temperature, the number of layers and their interactions at a 99% confidence level. Patou et al. [7] examined the influence of the consolidation process on the voids and mechanical properties of powdered and commingle glass/PPS (polyphenylene sulfide) laminates and found that increasing consolidation pressure resulted in a more homogeneous microstructure and a reduction in porosity, which improved the mechanical properties. Feng et al. [8] studied the effects of injection molding parameters on the microstructure and mechanical properties of a PE (polyethylene) composite reinforced with long glass fibers. They found that increasing injection pressure and melt temperature resulted in a more uniform distribution of fibers and improved mechanical properties. Overall, these studies showed that consolidation is highly dependent on the process parameters applied during the manufacturing of the composite part.

The emergence of new CFRTP (carbon fiber reinforced thermoplastic polymers) manufacturing technologies makes it necessary to continue investigating consolidation parameters, especially when these technologies differ from the standard due to their continuous application—such as glide forming [9,10,11,12] and Continuous Compression Molding [13,14,15]).

GF (glide forming) is an advanced automated manufacturing technology currently under development and is specifically tailored for crafting slender structural components with intricate double curvature profiles using composite materials. This innovative technique addresses a critical industry need—the efficient production of complex geometries such as stringers, wing reinforcements and stabilizer components, all of which pose significant manufacturing challenges due to their lengthy and curved geometries. Traditional methods often fall short when it comes to achieving high production rates for these parts. What sets the GF process apart are its key advantages, not the least being its remarkable flexibility. By employing a flexible nozzle concept and localized heating, it can seamlessly adapt to create components with varying cross-sectional shapes, thus eliminating the need for extensive tooling changes and resulting in substantial time and cost savings. Furthermore, the process boasts high levels of automation, reducing labor demands, and its environmentally friendly approach, which eschews the necessity for heated tools, positions it as a cost-effective and sustainable manufacturing solution compared to alternatives.

Likewise, CCM (continuous compression molding) is also a manufacturing technology currently undergoing development to produce CFRTP components. This process involves feeding a continuous laminate into a compression molding machine that contains a series of heated molds or dies. The material is heated above its melting point and then compressed consecutively in different stages as it passes through the machine until the final geometry is achieved. CCM is a highly efficient process for producing large quantities of constant section components with consistent quality and dimensional accuracy. This process has many advantages over other manufacturing processes for CFRTP, such as stamp forming or hot forming. CCM can produce large components, and its production rate is relatively high. Moreover, the process can be automated, making it cost-effective for high-volume production. Despite these advantages, CCM has some limitations, such as high initial tooling costs and limited flexibility for small batch production.

To address the lack of studies around these emerging technologies, a statistical study was proposed aimed at identifying the optimal process parameters—temperature, pressure and time (forming speed)—for achieving optimal consolidation levels using continuous manufacturing technologies in LM-PAEK reinforced with carbon fibers.

## 2. Materials and Methods

The experiment was based on the most commonly used manufacturing processes such as stamp forming [16,17,18], hot press forming [19] or sheet forming processes [20]. The parameters were adjusted to meet the conditions of continuous production processes, especially for the glide forming.

The statistical analysis was conducted following an experimental sequence based on a 3-factor Box–Behnken design experiment [21,22,23] that led to two RSMs (response surface models): one for each control variable. The RSMs were assessed using an ANOVA (analysis of variance) analysis with a confidence level of 90%. These models could be used to implement numerical post-processing models to predict forming results at macroscale levels. Similarly, they could be used directly as a guide to fine-tune a specific manufacturing process.

### 2.1. Material and Samples

The experiment was conducted using unidirectional-carbon-fiber-reinforced LM-PAEK composite from Toray (Toray Cetex TC1225, T700, 145 gsm FAW, 34 wt% RC). The key properties of the material are presented in Table 1.

**Figure 1:**
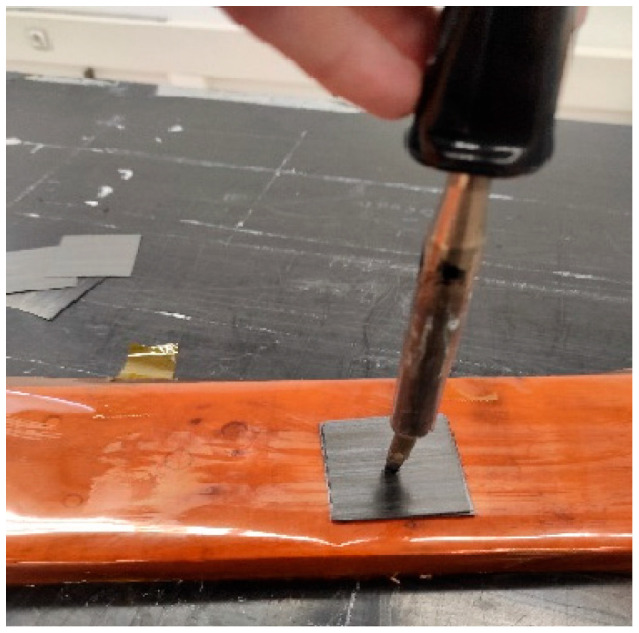
Ply-by-ply point-welding process.

The samples utilized in the experiment consisted of 25 mm × 25 mm laminates with a symmetric ply sequence [(0,90)_5_]_s_, point-welded in the center ply by ply (see Figure 1). Each laminate was equipped with a J-type thermocouple, which was positioned on the middle ply to monitor and control the temperature.

### 2.2. Experiment Setup Description

**Figure 2:**
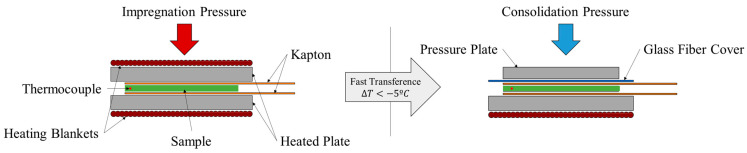
Experimental rig scheme. (**Left**): Stage 1 equivalent to the heating and impregnation phases of the glide forming process. (**Right**): Stage 2 equivalent to the consolidation phase of the glide forming process.

When manufacturing thermoplastic composite material components, three phases (pre-heating, impregnation and consolidation) are distinguished during the forming as described in reference [12]. They proposed to use this dynamic process in a static experimental setup which separates the whole process into two stages:

1. Pre-heating and impregnation/forming phases;
2. Consolidation phase;

Based on this concept, a special testing rig involving two presses adapted to each defined stage was designed (see Figure 2). Stage 1 press consisted of two 50 mm × 50 mm heating plates that were pressed using a 25 mm diameter pneumatic cylinder. This press simulated the application of pressure during the impregnation phase, where the molten material is shaped, and the intimate contact between the different plies is increased. The pressure application in this phase is less than that in the cooling phase. Stage 2 press consisted of a lower heating plate that simulated the heated tool and a nonheated upper plate that simulated the pressing skid (counter mold). Both plates had dimensions of 50 mm × 50 mm. The pressure was exerted by a 50 mm diameter pneumatic cylinder. The samples were wrapped in Kapton film to avoid the matrix sticking to the plates and a thermocouple was embedded in the middle ply of the sample to monitor the temperature during the test.

The experiment started by introducing the laminate into stage 1 press and heating the material. The heating time is equivalent to the actual heating time of the forming process. Once the temperature on the thermocouple reached the target value, the impregnation pressure was then applied for the corresponding duration of the stage in the real process. After this period, the samples were rapidly transferred to stage 2 press, where they were subjected to consolidation pressure for the designated time in the experimental sequence. To deem the sample acceptable, the temperature drops during the transfer had to be less than 5 °C.

#### 2.2.1. Typical Process Parameters

Previous studies, such as those conducted by Khan M.A. et al. [24] and Brooks R.A. et al. [17], established that, among all the parameters that influence the consolidation of a laminate, there were three separately controllable parameters, which were key for forming processes.

- Forming temperature: The material supplier typically provided the recommended range for forming temperatures. For TC1225/T700 [25], this range is normally between 320 °C and 400 °C.
- Consolidation pressure: The consolidation pressure range could vary depending on the manufacturing process. From previous experiences with high-rate applications, good consolidation values were achieved in a range of pressures between 0.2 and 2 MPa [12].
- Time: Time is an indirect measure of the forming speed. It was feasible to approximate the forming speed by considering a standard length for the forming skids (counter molds) used in glide forming. Thus, for the impregnation/forming phase-assuming a skid length of 200 mm and forming speeds between 1 mm/s and 10 mm/s—forming equivalent time varied from 20 s to 200 s. For the consolidation phase, the equivalent time considering a 100 mm skid varied from 10 s to 100 s.

Other parameters, such as the number of plies, impregnation pressure and tool temperature, may also impact the forming process but were kept constant in this study to reduce the number of variables. The number of plies was set to 20, the impregnation pressure to 4 kPa, and the tool temperature to 250 °C. These values were selected based on previous experiences with the glide forming process and material supplier indications.

- Number of plies: The number of plies in the laminate, a key parameter in the design of the component, is typically within a range of 5 to 20 for secondary structures. However, in certain cases, local reinforcements may increase the number of plies up to 50. For the purposes of this study, a laminate with 20 plies was chosen.
- Impregnation pressure: Impregnation pressure, applied during the forming phase, is utilized to secure the cohesion of the plies while the material is at the appropriate forming temperature. This results in a greater degree of intimate contact among the plies and ensures that each fiber is fully impregnated by the thermoplastic matrix. Additionally, impregnation pressure is employed in shaping the component. Based on previous research, an appropriate value for this pressure was determined to be 4 kPa.
- Tool temperature: Tool temperature, which is typically below melting point and above the crystallization temperature, was set to 250 °C for this study.

#### 2.2.2. Experimental Variables and Characterization

The consolidation of the component, measured by the porosity level and thickness, was studied to evaluate the results and identify the optimal process settings.

- Porosity: Porosity was quantified using the A-scan method via MUPE (manual ultrasound portable equipment). The transducer used was a mono-element straight beam transducer with a delay of 8.178 and a speed setting of 2713 m/s. The analysis involved comparing the sample attenuation to a reference standard. Micrographics were also utilized to provide a detailed examination of select samples. The methodology employed was in accordance with the Airbus AITM (Airbus Industries Test Methods) 6-4005 standard [26].
- Thickness: Thickness was measured using a micrometer and compared to the nominal thickness of a consolidated ply. The measured nominal thickness of the raw material was 0.170 mm.

### 2.3. Experiment Setup Description

A total of 19 samples were tested in accordance with the randomized experimental design. The study utilized the 3-parameter Box–Behnken design for 12 of the samples, which were set with varying parameters. Three samples were designated as the central point (0, 0, 0) for the purpose of evaluating the normal dispersion and repeatability of the experimental data. Additionally, four extra points were randomly selected to validate the models.

Randomization of the experiment design was crucial in ensuring the independence of sample conditions, as it prevented any potential influence on subsequent samples. This randomness provided a basis for drawing reliable conclusions from the test results, thus avoiding any ambiguities. The experimental factor ranges and the experimental sequence are presented in Table 2 and Table 3, respectively.

The results of the experiment were processed to determine the multiple response surface for each evaluation parameter. The multiple response surface provided a mathematical representation of the relationship between the various parameters and the evaluation criteria. While this approach alone may not provide highly accurate predictions, it serves as a useful and efficient means of obtaining a preliminary approximation. Further experimentation is required to validate the results and to enhance the level of accuracy of the predictions.

### 2.4. Evaluation of the Models

The statistical significance of the models obtained from the experimental results were determined using Analysis of Variance (ANOVA) with a confidence level of 90%. ANOVA is a powerful tool for evaluating the relationship between control variables and the observable response.

As per references [20,21,22], the change in response due to variations in control variables is known as the effect of these variables, and the ANOVA results provide adequate information to determine the validity of a model in explaining this relationship. To be considered statistically significant, the *p*-value of the coefficient must be less than the set confidence level, which was 0.1 in this case.

## 3. Results

The following section presents the results of the 19 runs, along with the regression models, their ANOVAs and micrographics. A summary of the results is provided in Table 3. The thickness results were obtained by taking three measurements per sample, which were then normalized and averaged. Normalization was performed to facilitate the applicability of the model to laminates with different thicknesses and sequences. A thickness of 3.40 mm, equivalent to 20 layers of 0.17 mm each, was considered.

The porosity results were obtained through the measurement of the amplitude of the backwall echo, using the protocol specified in the AITM-6-4005 [26] regulations. Three measurements were taken for each sample and the average was calculated. The amplitude of the backwall echo is indicative of the level of porosity of the material. The greater the amplitude, the lower the presence of pores and voids in the component.

### 3.1. Thickness Model

The thickness response surface is represented by Equation (1), with parameters listed in Table 4. The model comprises a quadratic term (PQ) of temperature, the interaction terms (TWI) between pressure and time, the linear terms (FO) of temperature, pressure and time and a constant.

TRS = β_0_ + β_1_·T + β_2_·P + β_3_·t + β_4_·P·t + β_5_·T^2^ (1)

where TRS is the thickness response surface, β_i_ are the model coefficients, T is the forming temperature, P is the consolidation pressure, and t is the equivalent time.

#### 3.1.1. Analysis of Variance

Table 4 displays the *p*-value of each coefficient in the thickness model. The *p*-value of the overall model (0.0000) is below the confidence level (0.1), thereby enabling the rejection of the null hypothesis. The null hypothesis states that there is no relationship between the control variables (temperature, consolidation pressure and equivalent time) and the dependent variable (thickness). This result confirms that the proposed quadratic model with interaction and linear terms accurately explains the relationship between thickness, temperature, pressure and time.

The effect of each coefficient in the equation was analyzed, and it was found that the squared term of temperature, linear term of temperature and the interaction term of pressure and time had a significant impact on the thickness results. On the other hand, the linear terms of pressure and time were found to be insignificant for the model but were retained because of the significant impact their interaction had on the final thickness of the component.

#### 3.1.2. Response Surface Results

The study of the influence of three parameters on component manufacturing was conducted by setting the equivalent time as a constant variable.

**Figure 3:**
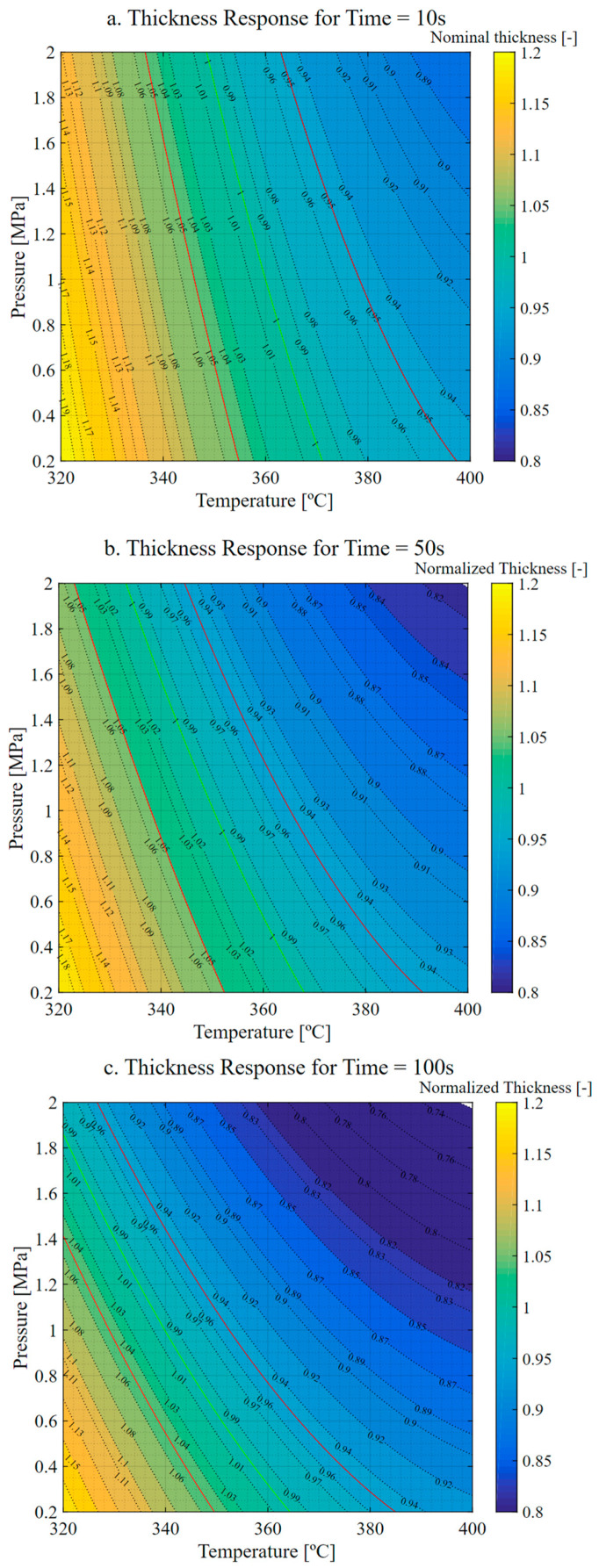
Thickness response. (**a**) Response for t = 10 s. (**b**) Response for t = 50 s. (**c**) Response for t = 100 s. Green indicates nominal thickness. Red indicates thickness tolerances (±5%).

The response surface results, shown in Figure 3, display three sections corresponding to equivalent times of 10, 50 and 100 s. The trend observed from the plots is that the maximum thickness values are achieved at low temperature and pressure, while the minimum thickness values are observed at high temperature and pressure. Moreover, the green iso-curve representing the nominal design thickness shifts towards lower temperature and pressure requirements as the equivalent time increases.

The relationship between temperature, pressure and the viscosity of the matrix (as described in references [23,24,27]) is the basis for explaining the observed behavior. As temperature increases, the viscosity of the matrix decreases, increasing matrix mobility and reducing the amount of pressure and time required. However, reducing viscosity could boost the matrix flowing outwards from the component if the applied pressure or the application time is excessive. These effects could lead to a thinner component as the matrix creeps more easily.

#### 3.1.3. Diagnostic Model

**Figure 4:**
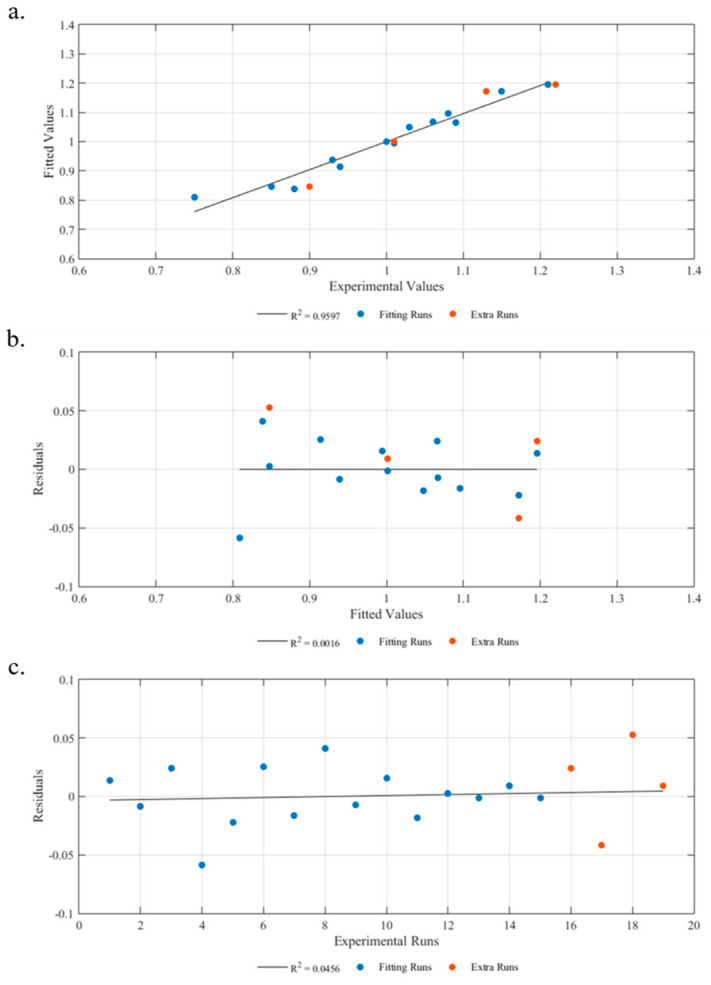
Thickness model diagnostic. (**a**) Fitted values vs. experimental values. (**b**) Residuals vs. fitted values. (**c**) Residuals vs. experimental runs.

Figure 4a presents a comparison between the experimental values and the predictions generated by the statistical model. The results clearly demonstrate that the equation derived from the model provides accurate predictions of the part’s thickness as a function of the control variables under the testing conditions. Table 4 further supports this conclusion, as the high R^2^ (95.82%) and adjR^2^ (93.49%) values indicate that the model is able to predict the response with over 90% accuracy.

A residual analysis, depicted in Figure 4b, reveals no discernible pattern, suggesting a homogeneous distribution of residuals and lending credibility to the model. To further evaluate the statistical significance of the model, the residuals were analyzed against the number of runs. As depicted in Figure 4c, there is no significant correlation between the residuals and the number of runs, which eliminates any potential relationship between the number of tests and the results obtained.

### 3.2. Backwall Echo Model

The backwall echo response surface revealed a more complex relationship between the independent variables and the consolidation level of the final part, thus suggesting logarithmic dependencies. The model indicated that the interaction of pressure and time, as well as the linear terms of temperature and pressure, were the most significant parameters within the model. Equation (2) represents the model in its logarithmic and exponential forms.

BRS = P^β2^·T ^β3^·exp[β_0_ + β_1_·T + β_4_·log(P)·log(t)] (2)

where BRS is the backwall echo response surface, β_i_ are the model coefficients, T is the forming temperature, P is the consolidation pressure, and t is the equivalent time.

#### 3.2.1. Analysis of Variance

The variance of the model was analyzed using a 90% confidence interval, following the methodology described in a previous section. The results of the ANOVA, summarized in Table 5, reveal that the global *p*-value (0.0003) is significantly lower than the confidence interval. This result supports the rejection of the null hypothesis, indicating the feasibility of a relationship between the amplitude backwall echo and the control parameters.

Upon analyzing the effect of each coefficient, the amplitude backwall echo was found to be highly sensitive to variations in temperature and pressure. In contrast, the interaction between the logarithm of pressure and temperature exhibited lower significance. The logarithm of pressure did not exhibit a *p*-value lower than the confidence interval, thus rendering it statistically insignificant.

#### 3.2.2. Response Surface Results

**Figure 5:**
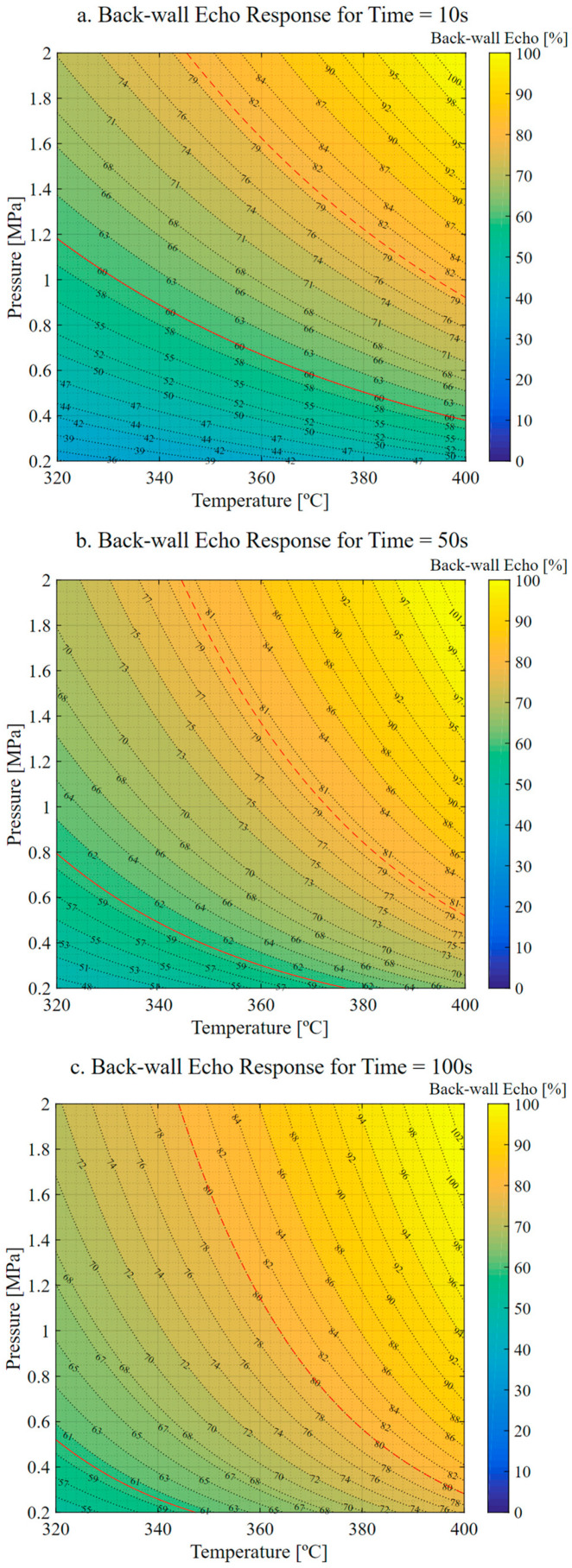
Backwall echo response. (**a**) Response for t = 10 s. (**b**) Response for t = 50 s. (**c**) Response for t = 100 s. Dashed red line is the minimum amplitude of backwall echo (BWE) allowed for parts with a nominal thickness greater than 5 mm. Solid red line is the minimum BWE allowed for parts with a nominal thickness less than 5 mm.

Figure 5 presents the porosity response surface against the control parameters. The two iso-curves highlighted in the figure represent the minimum acceptable amplitude of backwall echo according to reference [26]. The solid red line indicates the limit for thicknesses less than 5 mm, while the dashed line marks the minimum measurement for thicknesses greater than 5 mm.

The results show that the highest degree of consolidation occurs at the upper right corner of the surface, corresponding to the maximum temperature and pressure. Conversely, the minimum amplitude of backwall echo is observed at the lower left corner, where temperature and pressure are at their lowest values. As the equivalent time increases, the backwall amplitude becomes higher for lower temperatures and pressures.

These findings suggest a correlation between the control variables of thickness and porosity. The highest amplitude of the backwall echo is recorded in the case of minimum thickness. This correlation may be attributed to the creep behavior of the material, which helps in evacuating the air trapped between the plies and molecules, leading to a more compact structure with reduced porosity. This relationship agrees with Darcy’s law that describes the flow of a fluid through a porous medium (applicable to laminar fluid flow through a porous media at low flow rates).

#### 3.2.3. Diagnostic Model

**Figure 6:**
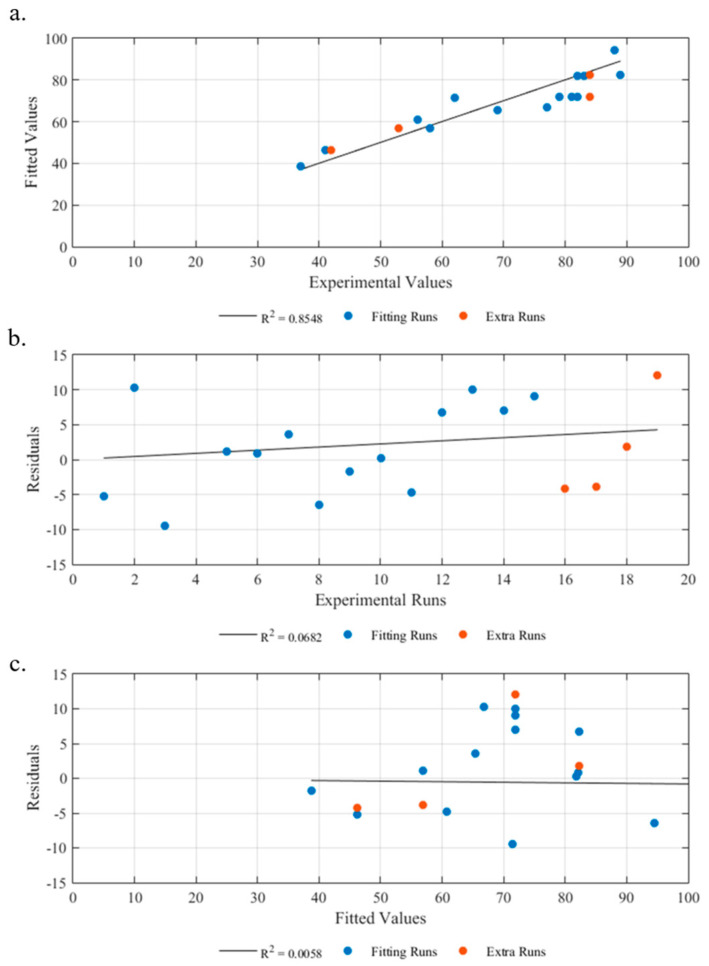
Backwall echo model diagnostic. (**a**) Fitted values vs. experimental values. (**b**) Residuals vs. fitted values. (**c**) Residuals vs. experimental runs.

The results of the model diagnosis (Figure 6a) indicate a low correlation between the predicted values and the experimental ones, as evidenced by the R^2^ (0.8548) and adjR^2^ (0.7968) values, which are lower than those of the thickness model. Despite this, the model still explains approximately 85% of the observed variation in the data. Further analysis of the residuals and the fitted values (Figure 6b), as well as the residuals and the experimental runs (Figure 6c), failed to reveal any significant relationships, suggesting the absence of any association between these variables.

### 3.3. Micrographics

**Figure 7:**
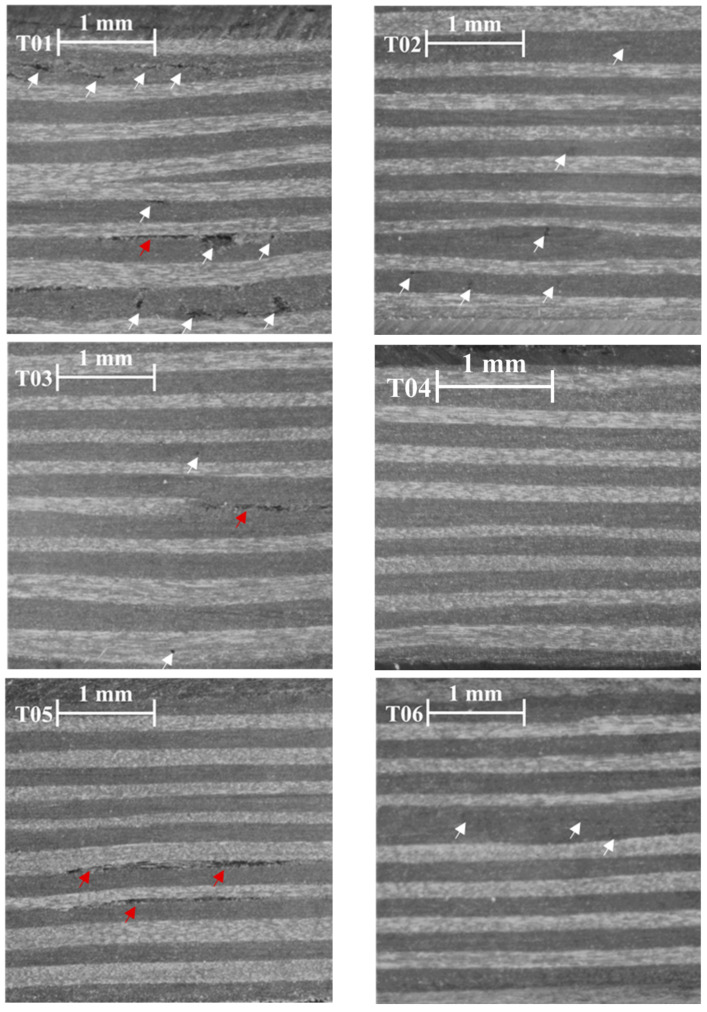

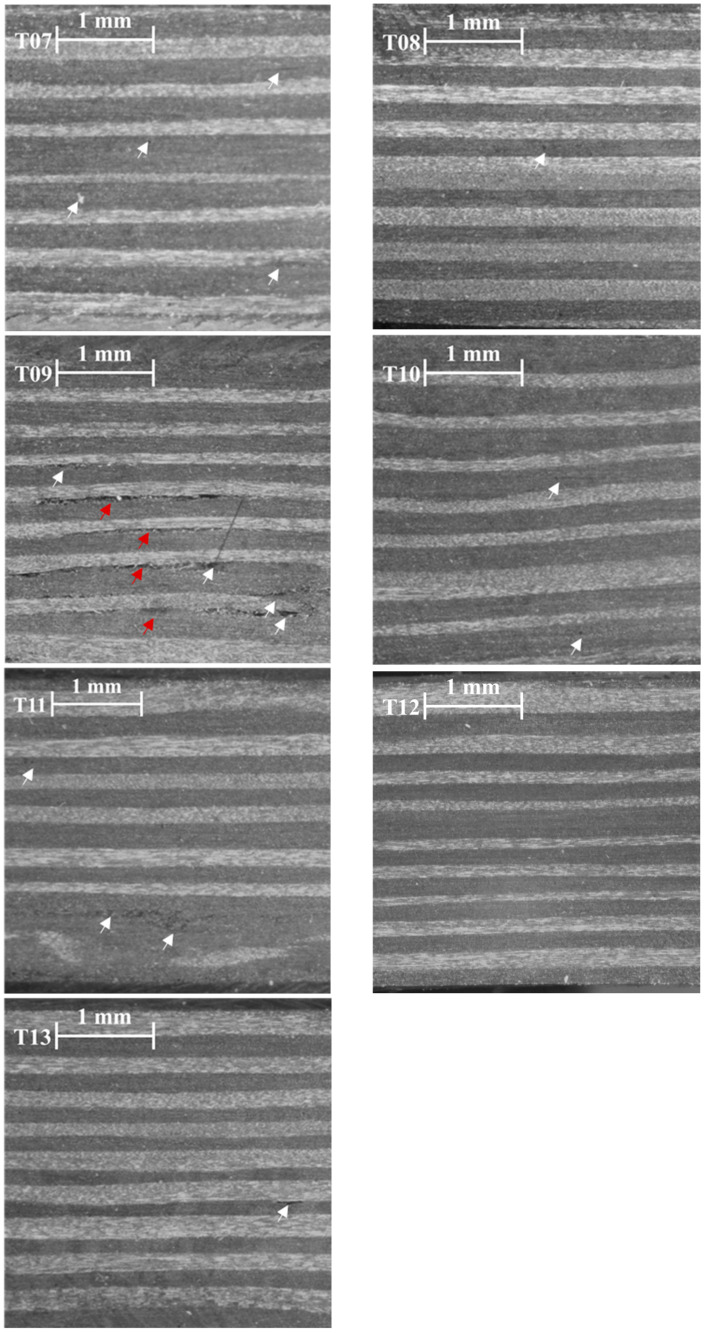
Micrographics of the samples used for the model fitting. White indicates voids. Red indicates delamination.

Figure 7 summarizes the microstructural examination of the samples generated from the Box–Behnken experimental design. The results of the models are illustrated through these micrographics. For example, sample T04 was found to be the thinnest among the samples and displayed a homogeneous distribution of the layers without any visible pores or defects. The forming parameters of T = 400 °C, P = 2 MPa and t = 50 s resulted in a predicted thickness of 0.81 and 85% according to the model, while the experimental results were 0.75 and 85%. These values corresponded to the maximum level of amplitude of the backwall echo and the minimum level of thickness, positioning the test point in the upper right corner of both graphs. Sample T13 achieved a thickness closest to the nominal value. Under the conditions of T = 350 °C, P = 1 MPa and t = 50 s, the model predicted a thickness of 1.00 and 81%. In contrast, sample T09 showed the poorest results in the model, as evidenced by the micrograph, which reveals significant air bubbles trapped between the layers and delamination.

## 4. Discussion

### 4.1. Relationship between Thickness and Porosity

**Figure 8:**
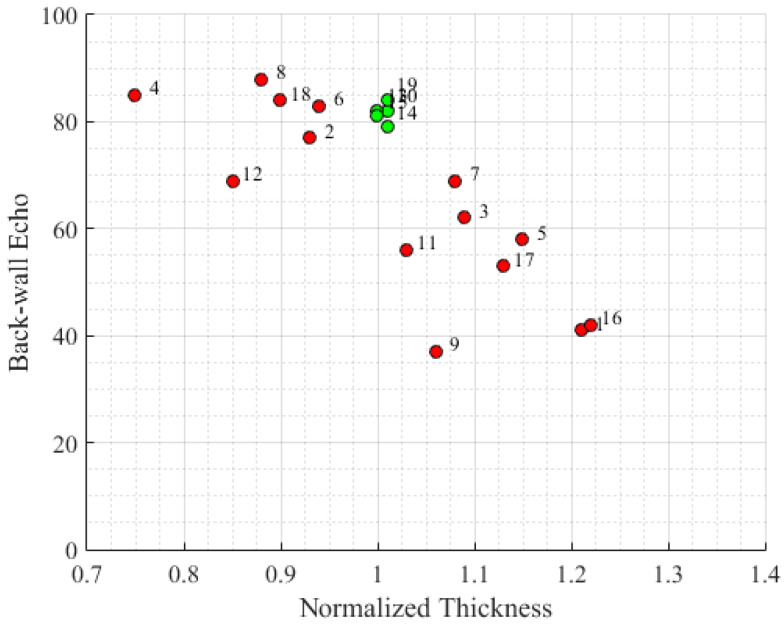
Amplitude of the backwall echo vs. normalized thickness. Green indicates good quality of the manufactured samples with values withing tolerances. Red indicates values outside tolerances.

As previously reported, the thickness and porosity level of a manufactured part are strongly correlated. Figure 8 clearly illustrates this relationship, with a decrease in the amplitude of the backwall echo observed as the thickness increases, in accordance with Darcy’s law.

The observed effects are in line with expectations, as a reduced thickness leads to increased material flow, enabling the filling of all existing pores within the matrix, thereby reaching higher levels of porosity. This effect underscores the significance of controlling the parameters, as conditions favoring the nominal thickness are found to decrease the amplitude of the backwall echo.

**Figure 9:**
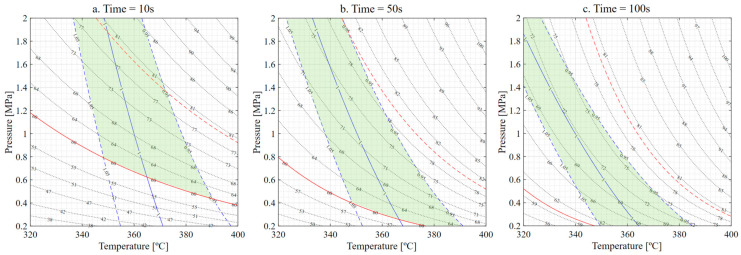
Amplitude of the backwall echo vs. normalized thickness. (**a**) Time slice of 10 s. (**b**) Time slice of 50 s. (**c**) Time slice of 100 s. Green indicates good quality of the manufactured samples. Dashed blue lines represent nominal thickness ± 5%. Solid blue lines indicate nominal thickness. Dashed red lines are the minimum amplitude of the backwall echo (BWE) allowed for parts with a nominal thickness greater than 5 mm. Solid red lines are the minimum BWE allowed for parts with a nominal thickness less than 5 mm.

The superimposed graphs of the thickness and amplitude of the backwall echo are presented in Figure 9. In this figure, the manufacturing conditions that yield components meeting both criteria (thickness and amplitude of the backwall echo) are highlighted in green. The first graph depicts a consolidation time of 10 s, corresponding to a velocity of 10 mm/s. This velocity condition is the only one capable of achieving sufficient consolidation levels for components exceeding 5 mm in thickness. However, it is worth noting that such high velocity necessitates elevated temperature and pressure values to achieve favorable thickness outcomes.

Conversely, the other end of the spectrum is represented by the figure corresponding to 100 s, i.e., equivalent to a velocity of 1 mm/s. As indicated by the outcomes, the slower material flow due to the reduced velocity leads to lower temperature and pressure requirements as the material has more time to flow. Surprisingly, this prolonged flow time demonstrates a counterproductive impact, resulting in higher porosity compared to the other two time frames tested.

Another noteworthy trend that is discernible from the figures is that lower pressure expands the range of temperatures that yield thickness values within tolerance. On the other hand, as pressure increases, temperature control becomes increasingly critical, given the reduced thermal range.

In light of these findings, it is evident that the consolidation, thickness and forming conditions wield a substantial influence on the resulting composite material properties. The interaction between parameters such as consolidation time, temperature and pressure plays a decisive role in determining the porosity and thickness outcomes of the manufactured parts. The delicate balance between these parameters is crucial to striking the right balance between sufficient material flow for pore filling and avoiding excessive porosity.

Furthermore, the relationship between temperature, pressure and time underscores the trade-offs inherent in the forming process. While higher velocities facilitated quicker production and reduced cycle times, they necessitated higher temperatures and pressures to compensate for the limited material flow duration. On the other hand, lower velocities offer extended flow periods, allowing for decreased temperature and pressure requirements, but at the cost of potentially reduced material quality due to increased porosity.

### 4.2. Process Optimization

One of the objectives of the present study was to establish the parameters for the glide forming process adapted to thermoplastic composite materials. Similar to other manufacturing processes, lower values of the controllable parameters are typically preferred due to ease and cost considerations. High-temperature and high-pressure processes pose challenges because of the lack of auxiliary materials and complex heating systems, respectively. Thus, the aim was to find the minimum pressure and temperature that can meet the quality requirements of the parts being manufactured while also maintaining a high rate of production to remain commercially competitive.

**Figure 10:**
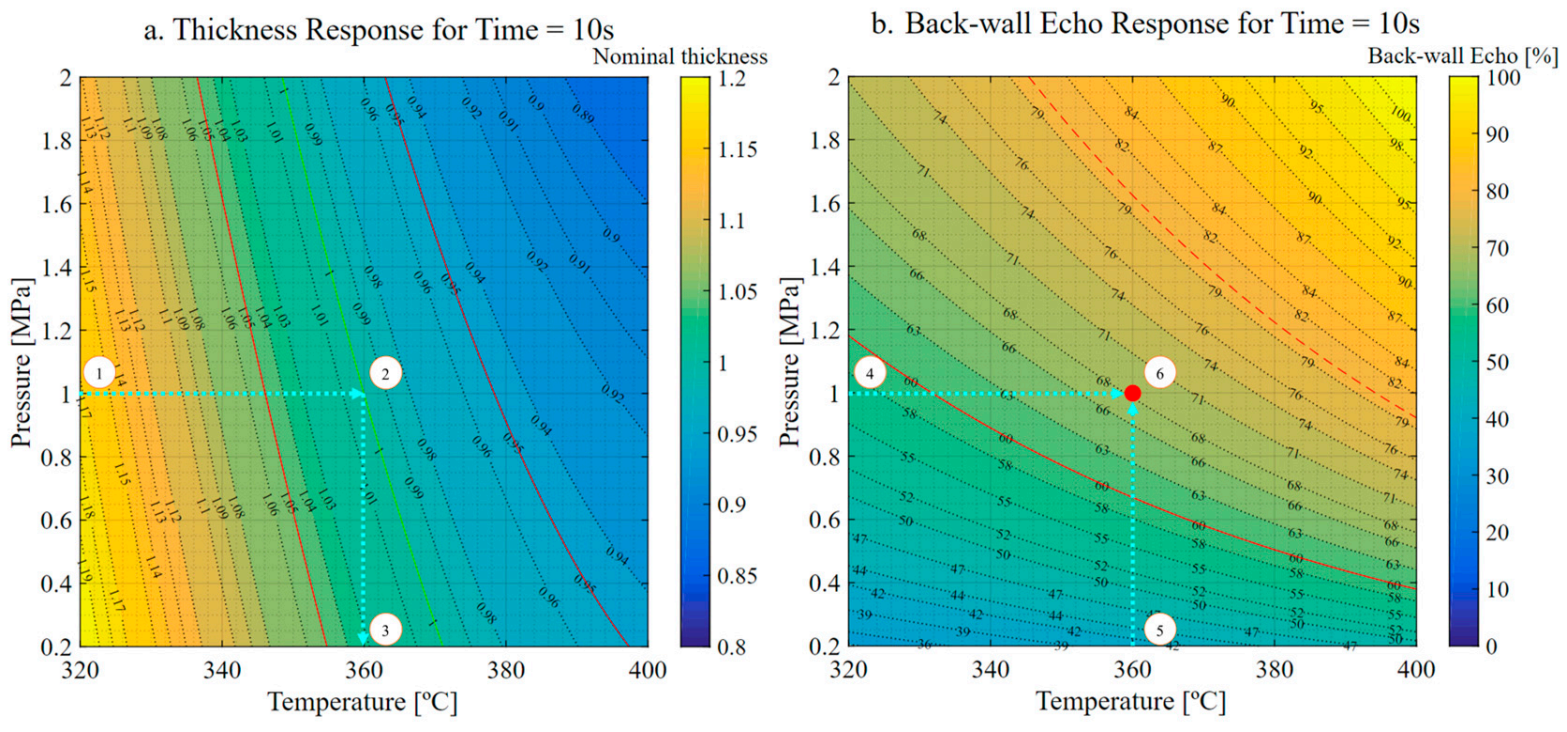
Process optimization sequence. 1. Maximum pressure available is selected. 2. Nominal thickness is pointed. 3. Temperature required is obtained. 4. Maximum pressure available is selected. 5. Temperature required is selected. 6. The consolidation level predicted is pointed where pressure and temperature intersect. In the left image, green solid line represents nominal thickness. Red solid lines indicated nominal thickness ± 5%. In the right image, dashed red line indicates the minimum amplitude of the back-wall echo (BWE) allowed for parts with a nominal thickness greater than 5 mm. Solid red line is the minimum BWE allowed for parts with a nominal thickness less than 5 mm. Red dot marks the predicted consolidation level.

The presented models can assist this determination by considering the limitations of tooling and auxiliary materials. An example of the use of these models is illustrated in Figure 10. In this scenario, a part with 15 plies (2.5 mm) must be manufactured, with strict control over its thickness and a requirement for an amplitude of the backwall echo greater than 60%. The machine has a maximum pressure limitation of 1 MPa, and the required production rate is 10 mm/s.

With this information, the models were used to set the production parameters focused on production rate and thickness. The 10 s section plots were selected to meet the manufacturing rate. This rate requires a forming temperature of 359 °C and a pressure of 1 MPa, resulting in a normalized thickness of 1.0 and an amplitude of backwall echo of 68%.

## 5. Conclusions

In conclusion, the study suggested that continuous consolidation manufacturing technologies, such as glide forming, could be adapted to thermoplastic composite materials, but the process parameters must be carefully controlled to ensure the quality of the final part. The results of this study provide important information for the industrial application of the glide forming technology to thermoplastic composite materials. The ANOVA analysis allowed us to establish the significant manufacturing parameters affecting the quality of the laminate, leading to two response surface models. These models could be used as a post-processing tool to predict the thickness and consolidation level when simulating the manufacture of a component on macroscale levels. Further research is needed to optimize the process parameters and to study the effects for other thermoplastic composite materials.

## Figures and Tables

**Table 1:** Material properties.

Property	TC1225/T700
Type of composite	Unidirectional Prepreg
Type of reinforcement	T700
Fiber areal weight (FAW)	$145\;g/m^{2}$
Resin content by weight (RC)	34%
Consolidated ply thickness (CPT)	0.185 ± 0.016 mm

**Table 2:** Values for the three factors.

Factor	Description	Low (−1)	Midpoint (0)	High (+1)
A	Temperature	320 °C	350 °C	400 °C
B	Consolidation Pressure	0.2 MPa	1 MPa	2 MPa
C	Consolidation Time	10 s	50 s	100 s

**Table 3:** Randomize experimental sequence and results.

Sample	Order	Factor			Normalized Thickness	Backwall Echo Amplitude (%)
		A	B	C		
T01	19	−1	−1	0	1.21 ± 0.014	41 ± 1.132
T02	9	1	−1	0	0.93 ± 0.005	77 ± 1.729
T03	1	−1	1	0	1.09 ± 0.017	62 ± 1.729
T04	18	1	1	0	0.75 ± 0.006	85 ± 0.653
T05	13	−1	0	−1	1.15 ± 0.006	58 ± 2.356
T06	10	1	0	−1	0.94 ± 0.013	83 ± 2.848
T07	15	−1	0	1	1.08 ± 0.013	69 ± 2.848
T08	5	1	0	1	0.88 ± 0.006	88 ± 2.848
T09	7	0	−1	−1	1.06 ± 0.006	37 ± 1.960
T10	8	0	1	−1	1.01 ± 0.006	82 ± 2.356
T11	14	0	−1	1	1.03 ± 0.006	56 ± 4.573
T12	2	0	1	1	0.85 ± 0.006	89 ± 1.729
T13	16	0	0	0	1.00 ± 0.003	82 ± 1.802
T14	3	0	0	0	1.01 ± 0.003	79 ± 1.802
T15	6	0	0	0	1.00 ± 0.006	81 ± 1.820
T16	12	−1	−1	0	1.22 ± 0.006	42 ± 3.638
T17	17	−1	0	−1	1.13 ± 0.003	53 ± 5.696
T18	4	0	1	1	0.90 ± 0.006	84 ± 2.356
T19	11	0	0	0	1.01 ± 0.006	84 ± 1.820

**Table 4:** Thickness model. Regression analysis coefficients and ANOVA. Analysis performed on RStudio. FO: first order terms; TWI: interaction terms; PQ: pure quadratic terms; Df: degrees of freedom; Sum Sq: sum of squares; Mean Sq: mean squares.

	Type and Affected Parameters		Coefficients	*p*-Value	
β_0_	Constant		5.7084 × 10^00^	0.0024	
β_1_	Linear term of T		−2.2711 × 10^−02^	0.0153	
β_2_	Linear term of P		−3.2726 × 10^−02^	(0.1819)	
β_3_	Linear term of t		−5.3302 × 10^−05^	(0.9088)	
β_4_	Interaction term of P·t		−7.8929 × 10^−04^	0.0607	
β_5_	Squared term of T		2.7073 × 10^−05^	0.0298	
**R^2^**	0.9582				
**adjR^2^**	0.9349				
***p*** **-Value (fstats)**	0.0000				
	**Df**	**Sum Sq**	**Mean Sq**	**F Value**	**Pr (>F)**
**FO (T, P, t)**	3	0.1754	0.0587	64.9335	0.0000
**TWI (P, t)**	1	0.0042	0.0042	4.6602	0.0592
**PQ (T)**	1	0.0059	0.0059	6.6430	0.0298
**Residuals**	9	0.0081	0.0009		

**Table 5:** Backwall echo model. Regression analysis coefficients and ANOVA. Analysis performed on RStudio. FO: first order terms; TWI: interaction terms; PQ: pure quadratic terms; Df: degrees of freedom; Sum Sq: sum of squares; Mean Sq: mean squares.

	Type and Affected Parameters		Coefficients	*p*-Value	
β_0_	Constant		2.4528	0.0001	
β_1_	Linear term of T		0.0046	0.0017	
β_2_	Linear term of log(P)		0.5175	0.0102	
β_3_	Linear term of log(t)		0.0607	(0.1423)	
β _4_	Interaction term of log(P)·log(t)		−0.0838	0.0823	
**R^2^**	0.8548				
**adjR^2^**	0.7968				
***p*** **-Value (fstats)**	0.0003				
	**Df**	**Sum Sq**	**Mean Sq**	**F Value**	**Pr (>F)**
**FO (T, log(P), log(t))**	3	0.8534	0.2844	18.3873	0.0002
**TWI (log(P), log(t))**	1	0.0576	0.0576	3.7284	0.0823
**Residuals**	10	0.1547	0.1547		

## Data Availability

Data are only available upon reasonable request.

## References

[1] Biron M. (2018). Thermoplastic Composites, in Thermoplastic and Thermoplastic Composites.

[2] Steenkamer A., Sullivan J.L. (1998). On the recyclability of a cyclic thermoplastic composite material. Compos. Part B Eng..

[3] Pegoretti A. (2021). Towards sustainable structural composites: A review on the recycling of continuous-fiber-reinforced thermoplastics. Adv. Ind. Eng. Polym. Res..

[4] Martin I., Saenz del Castillo D., Fernandez A., Güemes A. (2020). Advanced thermoplastic composite manufacturing by in-situ consolidation: A review. J. Compos. Sci..

[5] Arquier R., Iliopoulos I., Réginier G., Miquelard-Garnier G. (2022). Consolidation of continuous-carbon-fiber-reinforced PAEK composites: A review. Mater. Today Commun..

[6] Colton J.M., Muzzy J., Birger S., Yang H., Norpoth L. (1992). Processing parameters for consolidating PEEK/carbon fibre (APC-2) composites. Polym. Compos..

[7] Patou J., Bonnaire R., De Luycker E., Bernhart G. (2019). Influence of consolidation process on voids and mechanical properties of powdered and commingled carbon/PPS laminates. Compos. Part. A Appl. Sci. Manuf..

[8] Liu F., Li T., Xu F., Li J., Jiang S. (2020). Microstructure, Tensile Property, and Surface Quality of Glass Fiber-Reinforced Polypropylene Parts by rapid heat cycle molding. Adv. Polym. Technol..

[9] Brufau J., Lorente A.S. (2017). System for Forming Stacks of Composite Materials. U.S. Patent.

[10] Brufau J. (2018). System for Forming Stacks of Composite Materials. U.S. Patent.

[11] Robins B.G., Berrios I., Redondo J.B., Rueda M.C.C. (2020). Heat Blankets Assembly for Forming a Composite Charge. U.S. Patent.

[12] Campos D., Brufau J., Biurrun M., Martín A. A Review of the A+ Glide Forming Manufacturing Technology and its Adaptation to CFRP Thermoplastic Composite Materials. Proceedings of the 5th International Conference & Exhibition on Thermoplastic Composites.

[13] Enoki S., Kojima K., Mizuno S., Katayama K., Tanaka K. (2014). High-speed compression molding of continuous carbon fiber reinforced polypropylene. WIT Trans. Built Environ..

[14] Piott F., Krämer A., Lück A., Hoffmann L., Mitschang P., Drummer D. (2021). Increasing the performance of continuous compression moulding by local pressure adaption. Adv. Manuf. Polym. Comppos. Sci..

[15] Christmann M., Medina L., Mitschang P. (2016). Effects of inhomogeneous temperature distribution on the impregnation process of the continuous compression molding technology. J. Thermoplast..

[16] Chih-Min M., Cheng-Tao Y., Bor-Wen C. (2014). Optimization of stamp forming process for thermoplastic composites. Res. J. Appl. Sci. Eng. Technol..

[17] Brooks R.A., Wang H., Zerong D., Jie X., Song Q., Liu H., Dear J.P., Li N. (2022). A review on stamp forming of continuous fibre-reinforced thermoplastics. Int. J. Lightweight Mater. Manuf..

[18] McCool R., Murphy A., Wilson R., Jiang Z., Price M., Butterfield J., Hornsby P. (2012). Thermoforming carbon fiber-reinforced thermoplastic composites. Proc. Inst. Mech. Eng. Part L J. Mater. Des. Appl..

[19] Tatsuno D., Yoneyyama T., Okamoto M. Hot press forming of thermoplastic CFRP sheets. Proceedings of the 17th International Conference on Metal Forming.

[20] Yanagimoto J., Ikeuchi K. (2012). Sheet forming process of carbon fiber reinforced plastics for lightweight parts. CIRP Annals Manuf. Tech..

[21] Tukey J.W. (1977). Exploratory Data Analysis.

[22] Kutner M., Nachtsheim C., Neter J., Li W. (2004). Applied Linear Statistical Models.

[23] Box G.E.P., Cox D.R. (1964). An analysis of transformations. J. R. Statist. Soc. B.

[24] Khan M.A., Mitschang P., Schledjewski R. (2010). Identification of some optimal parameters to achieve higher laminate quality through tape placement process. Adv. Polym. Technol..

[25] Toray Advanced Composites (2019). Toray Cetex TC1225 Product Data Sheet. https://www.toraytac.com/media/3bd72fac-0406-48e4-bfc4-2ffd2398ac0c/zipxIA/TAC/Documents/Data_sheets/Thermoplastic/UDtapes%2Cprepregsandlaminates/Toray-Cetex-TC1225_PAEK_PDS.pdf.

[26] (2018). Ultrasonic Pulse-Echo Inspection of Carbon Fibre Plastic.

[27] Deignan A., Figiel L., McCarthy M.A. (2018). Insights into complex rheological behaviour of carbon fibre/PEEK from a novel numerical methodology incorporating fibre friction and melt viscosity. Compos. Strct..

